# The Role of Mitophagy in Innate Immunity

**DOI:** 10.3389/fimmu.2018.01283

**Published:** 2018-06-05

**Authors:** Ilias Gkikas, Konstantinos Palikaras, Nektarios Tavernarakis

**Affiliations:** ^1^Institute of Molecular Biology and Biotechnology, Foundation for Research and Technology-Hellas, Heraklion, Greece; ^2^Department of Biology, University of Crete, Heraklion, Greece; ^3^Department of Basic Sciences, Faculty of Medicine, University of Crete, Heraklion, Greece

**Keywords:** autophagy, energy homeostasis, immunity, inflammation, metabolism, macrophages, mitochondria, mitophagy

## Abstract

Mitochondria are cellular organelles essential for multiple biological processes, including energy production, metabolites biosynthesis, cell death, and immunological responses among others. Recent advances in the field of immunology research reveal the pivotal role of energy metabolism in innate immune cells fate and function. Therefore, the maintenance of mitochondrial network integrity and activity is a prerequisite for immune system homeostasis. Mitochondrial selective autophagy, known as mitophagy, surveils mitochondrial population eliminating superfluous and/or impaired organelles and mediating cellular survival and viability in response to injury/trauma and infection. Defective removal of damaged mitochondria leads to hyperactivation of inflammatory signaling pathways and subsequently to chronic systemic inflammation and development of inflammatory diseases. Here, we review the molecular mechanisms of mitophagy and highlight its critical role in the innate immune system homeostasis.

## Introduction

The immune system is an intricate network of distinct cell types, tissues, and organs acting synergistically to protect the entire organism against the invasion of various pathogens, including bacteria, fungi, parasites, and viruses among others. The regulation of immune system is a multistep and complex process that classically includes signaling pathways initiated in the surface of immune cells and transmitted to the nucleus through a cascade of phosphorylation events. In turn, epigenetic, transcriptional, posttranscriptional, translational, and posttranslational modifications take place and influence several aspects of innate and adaptive immunity (1, 2). These molecular mechanisms are tightly coordinated and define the onset, the duration, and the magnitude of immune responses neutralizing foreign pathogenic microorganisms and resolving injury (3–6).

Recent evidence underlines the pivotal role of energy metabolism in the regulation of immunity. Mitochondria are dynamic organelles that modify their function, distribution, and structure in response to metabolic state of the cell (7). Proper mitochondrial function not only provides the required energy but also is essential for the establishment and the maintenance of immune cells phenotype and activity (8). Mitochondrial defects, characterized by cytoplasmic calcium elevation, increased reactive oxygen species (ROS) levels, and pronounced release of pro-apoptotic factors and mitochondrial DNA (mtDNA), are key stimulators of inflammatory response pathways (9). Inflammation is a cytoprotective response preserving tissue homeostasis and ensuring viability upon infection or injury (10). Innate immune cells, such as neurotrophils and macrophages, detect harmful stimuli and initiate the inflammatory signaling pathways. However, persistent and unresolved inflammation in metabolic tissues, such as adipose, liver, pancreas, and muscle, leads to the development and progression of several inflammatory pathologies, including atherosclerosis, type-2 diabetes, metabolic syndrome, and inflammatory bowel disease among others (11). The actions of macrophages in these ailments are highly appreciated (12). Inflammasomes are innate immune system receptors and sensors initiating inflammatory responses (13). Excessive mitochondrial dysfunction mediates inflammasome overstimulation in response to noxious stimuli, such as pathogens and cellular debris. In turn, caspase-1 is activated resulting in the generation of pro-inflammatory cytokines and promoting inflammatory cell death (14). Accumulating evidence interconnects impaired energy metabolism and inflammasome hyperstimulation (14–20). Therefore, repairing of mitochondrial functional deficiency or removal of damaged organelles might be beneficial against the undesired chronic systemic inflammation.

In this review, we focus on the role of mitophagy in innate immune system. We first describe the molecular pathways that govern mitophagy as well as its complex interplay with microbe selective autophagy, known as xenophagy. Furthermore, we discuss the essential role of energy metabolism and mitophagy in macrophage homeostasis and inflammasome stimulation. Better understanding of mitochondrial degradation mechanisms is a key requirement for the development of novel therapeutic interventions to tackle numerous pathologies in humans, including inflammatory diseases.

## Molecular Mechanisms of Mitochondrial Turnover

Cellular homeostasis is often undermined by misfolded and aggregated proteins, damaged organelles, and invading microbes, among others. As a consequence, cells have developed sophisticated quality control mechanisms that remove superfluous and/or damaged cytoplasmic components. Autophagy serves as such a clearance mechanism that is highly responsive to the nature of the stimulus. Based on the response, three different types of autophagy have been described including microautophagy, chaperon-mediated autophagy, and macroautophagy (21, 22). For the purpose of this review, the prominent type of macroautophagy (hereby referred to as autophagy) will be described. General autophagy machinery comprises autophagosome formation and maturation *via* irreversible steps of double-membrane vesicle nucleation and elongation. Mature double-membrane autophagosomes followed by induction of autophagic adaptor proteins can recognize, sequester, and enclose cellular cargo. Ultimately, fusion of the mature autophagosome with the lysosome mediates cargo degradation and recycling of intracellular material (23).

In the immune system, proper mitochondrial function is a prerequisite for inflammatory responses and host defense (24). Accumulation of damaged mitochondria results in excessive ROS production, elevated cytoplasmic calcium levels, and mtDNA release to the cytosol, which in turn triggers inflammasome activation (25–27). Aberrant inflammatory responses have been associated with the development of several autoimmune diseases. Therefore, targeting damaged mitochondria for degradation could be a promising therapeutic strategy against progressive inflammatory pathologies. The removal of damaged mitochondria required the activation of a selective autophagic process, known as mitophagy. Although the crosstalk between mitophagy mechanisms and host defense has been established only recently, a growing body of evidence supports the importance of their coordination.

**Figure 1:**
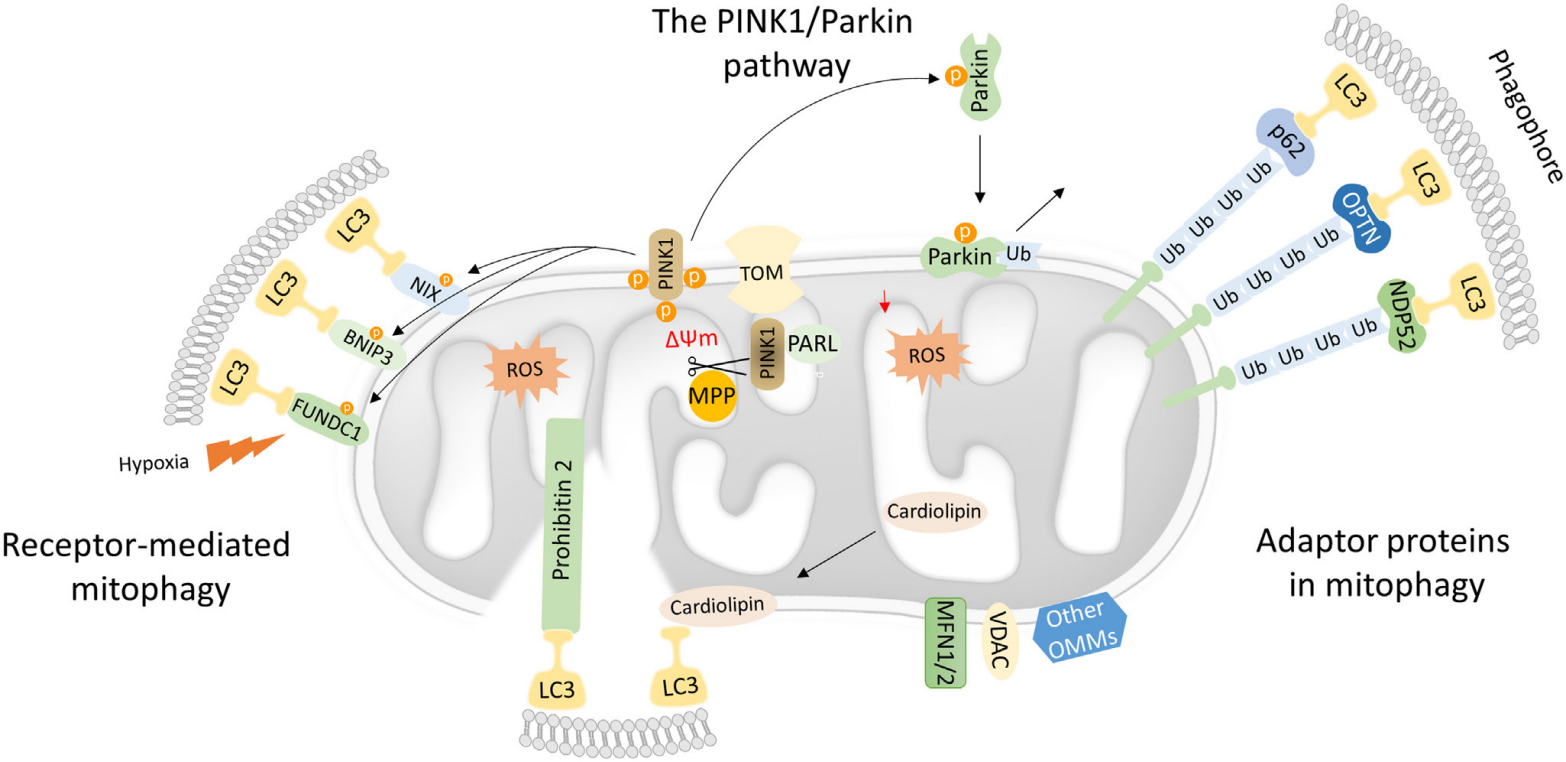
Mechanistic insights into mitophagy process. Dysfunctional mitochondria redirect PTEN-induced kinase 1 (PINK1) to the OMM while its proteolytic cleavage through mitochondrial processing peptidase (MPP) and presenilin-associated rhomboid-like (PARL) proteases is blocked. Concomitantly, PINK1 recruits Parkin through a series of modifications, such as phosphorylation of both Parkin and ubiquitin. In turn, Parkin triggers the polyubiquitination of various OMM proteins including voltage-dependent-anion-selective channel 1 (VDAC1) and MFN1/2. Polyubiquitinated proteins are recognized by several adaptor molecules, including p62, optineurin (OPTN), and NDP52, promoting their recognition by light chain 3 (LC3) and autophogosomal formation. Receptor-mediated mitophagy relies on various OMM proteins such as BNIP3, NIX, and FUN14 domain-containing protein 1 (FUNDC1). In addition, PHB2 and cardiolipin serve as inner mitochondrial membrane receptors in response to mitochondrial damage. Subsequently, PHB2 and cardiolipin are exposed to the cytosol mediating LC3 recruitment *via* their LIR motifs.

Following, recent evidence regarding the intricate role of mitophagy in inflammatory responses will be discussed in detail. The involvement of receptors and adaptors molecules is essential for mitophagy initiation and progression. Up to date, several mitochondrial proteins, located either in the outer (OMM) or the inner mitochondrial membrane (IMM), have been characterized as mitophagy receptors. Malfunctioning mitochondria are recognized by a microtubule-associated protein light chain 3 (LC3) in either ubiquitin-dependent or -independent manner (Figure 1). In turn, mitophagy receptors, which harbor an LC3-interacting region (LIR) motif, associate directly with LC3 and promote autophagosome formation (28).

### The PTEN-Induced Kinase 1 (PINK1)/Parkin Pathway

Mutations in the PINK1 and the E3-ubiquitin ligase (Parkin) were primary associated with Parkinson’s disease. Both PINK1 and Parkin are needed for proper mitochondrial function, although their role in mitochondrial turnover was appreciated only recently (29). Under physiological conditions, the transport of PINK1 preprotein into the IMM is followed by sequential proteolytic cleavage by the mitochondrial processing peptidase and presenilin-associated rhomboid-like protease (30–32). The remaining fragment of 52 kDa, which harbors the kinase domain of PINK1, is exposed to the cytosol until its final degradation by the proteasome. Under challenged conditions and loss of mitochondrial integrity, PINK1 fails to translocate to the IMM, and its proteolytic cleavage is blocked. Consequently, active PINK1 accumulates on the OMM though its interaction with the translocons of the outer mitochondrial membrane complex (TOM complex) (33). Then, PINK1 recruits Parkin through a circuit of modifications including phosphorylation of both Parkin and ubiquitin (34–38). Damaged mitochondria are tagged with active Parkin, which, in turn, mediates the polyubiqutination of several OMM proteins, including mitofusin 1 and 2 (MFN1/2), voltage-dependent-anion-selective channel 1, and mitochondrial import receptor subunit TOM20 homolog (TOMM20) among others (Figure 1) (39). In certain cases, Parkin-mediated polyubiquitination triggers the proteosomal degradation, as it has been documented for MFN1 and MFN2 (40, 41). As a consequence, mitochondrial fusion is prevented isolating damaged organelles form the healthy mitochondrial network. Thus, mitofusins degradation generates smaller mitochondria that can easily be sequestered by autophagosomal membranes.

### The Role of Adaptor Proteins in Mitochondrial Selective Autophagy

Following, Parkin-mediated ubiqutination of mitochondrial substrates, several adaptors proteins have been described to bind ubiquitin chains on the OMM promoting LC3 recruitment (42). Similar to the canonical autophagy mechanism, LC3 recognizes and interacts with the adaptor molecules through LIR motifs initiating autophagosomal formation. Numerous autophagy adaptors have been identified so far, including p62/sequestosome-1 (SQSTM1), optineurin (OPTN), next to BRCA1 gene 1 (NBR1), nuclear domain 10 protein 52 (NDP52), and TAX1 binding protein 1 (TAX1BP1) (Figure 1) (43).

While the autophagy adaptor p62/SQSTM1 binds ubiquitin chains on depolarized mitochondria and is essential for mitochondrial clustering in a Parkin-dependent manner, the exact role of p62/SQSTM1 in mitophagy has not been verified yet (44–46). Despite the similar kinetics of NDP52, TAX1BP1, and OPTN to dysfunctional mitochondria, cells lacking these adaptors fail to induce mitophagy (47–49). Particularly, loss of OPTN results in most prominent inhibition of mitophagy. Studies in mammalian cells demonstrate that PINK1-mediated recruitment of OPTN and NDP52 autophagy adaptors albeit Parkin was dispensable for mitophagy induction (45). Recent findings suggest that both NDP52 and OPTN are phopshorylated by the Tank-binding kinase 1 (TBK1) and, thereby, enhancing their binding affinity (48, 50–52). Interestingly, TBK1 is activated and phosphorylates OPTN in response to mitochondrial damage. Then, OPTN is recruited on the OMM-promoting mitochondrial elimination (49).

### Receptor-Mediated Mitophagy

Mitophagy receptors are commonly found on the outer and IMM. Certain OMM receptors of mitophagy have been identified, including BCL2 interacting protein 3 (BNIP3), Nip3-like protein X (NIX), and the FUN14 domain-containing protein 1 (FUNDC1) among others (43). Surprisingly, cardiolipin and prohibitin 2 (PHB2), which are located in IMM, have been also shown to serve as receptor proteins upon stress conditions (53–55). Mitophagy receptors contain LIR motifs that indicate their direct interaction with LC3 to promote the engulfment of defective mitochondria (Figure 1).

NIX contains a mitochondrial BH3 domain and interacts with LC3. NIX has been shown to mediate mitochondrial turnover during reticulocytes’ maturation (56). Specifically, NIX-mediated mitophagy relies on a specific motif within NIX cytoplasmic region, which acts as a signaling amplifier to launch additional mitophagic proteins (57). Under low oxygen levels, NIX transcriptional activity is regulated by hypoxia-inducible factor 1 (HIF1), while posttranslational phosphorylation at Ser81 drives mitochondrial clearance in ischemic stroke (58, 59). In addition, NIX phosphorylation at Ser34 and Ser35 residues surrounding LIR motif increases its binding affinity to LC3 (60). A recent study has also documented an alternative role of NIX-mediated mitochondrial quality control in human fibroblasts lacking PINK1 and Parkin (61, 62). This non-canonical regulation of mitophagy by NIX can give rise to novel therapeutic approaches for removal of malfunctioning mitochondria in Parkinson’s disease.

BNIP3 was also characterized as a BH3 protein on the OMM initially involved in cell death process (63). Despite its role in cell death, a potent role of BNIP3 in mitophagy has been reported. Specifically, its N-terminal LIR motif serves as a signaling platform for LC3-mediated mitochondrial sequestration through autophagosomes. Notably, sufficient LC3 binding is accompanied by BNIP3 phosphorylation at Ser17 and Ser24, proximal to the LIR motif (64–66). Surprisingly, BNIP3-deficient mammalian cells showed induction of PINK1 proteolysis and subsequently failed to promote mitophagy (67). Upon hypoxia, HIF1 triggers BNIP3 expression levels, which, in turn, inhibits cleavage of PINK1 proteolysis and promotes mitophagy (67). Depletion of DCT-1 the *Caenorhabditis elegans* homolog of both BNIP3 and NIX results in mitophagy inhibition suggesting a conserved role of autophagy receptors among species (68).

In mammalian cells, hypoxia promotes the binding of mitophagy receptor FUNDC1 to LC3 (69). Under normal oxygen levels, LC3 binding is perturbed due to phosphorylation of FUNDC1 at Tyr18 and Ser13 by Src and casein kinase II, respectively (70). In response to hypoxic conditions, phosphoglycerate mutase family member 5 phosphatase is activated and dephosphorylates FUNDC1 enabling its functional association with LC3 autophagosomal protein (71). Recently, it has been reported that FUNDC1 is a substrate of the serine/threonine-protein kinase unc-51-like kinase 1 (ULK1) (72). ULK1 translocates to damaged mitochondria and phosphorylates FUNDC1 at Ser17 triggering mitophagy in response to stress conditions (72). However, several homeostatic mechanisms have been evolved to regulate and fine-tune mitophagy during hypoxia (23, 73). FUNDC1-mediated mitophagy is block due to activation of receptor-interacting serine/threonine-protein kinase 3 followed by phosphorylation of FUNDC1 upon reperfusion injury (73). These results highlight the interplay between mitophagy and necroptosis to maintain cellular homeostasis during hypoxic conditions. Furthermore, mitochondrial E3-ubiquitin protein ligase 5 ubiquitinates FUNDC1 mediating its proteasomal degradation in response to hypoxia (23, 74).

Although the aforementioned receptors are located on the OMM, the possibility that an IMM protein could serve as a mitophagy receptor is not excluded. Toward this direction, PHB2 was recently characterized as an IMM mitophagy receptor (55). Particularly, it has been showed that Parkin-dependent loss of mitochondrial integrity and permeabilization of the OMM enhance the interaction between LC3 and PHB2, thereby promoting mitophagy. In addition, PHB2-mediated mitophagy is involved in selective clearance of paternal mitochondria in *C. elegans* embryos (55).

Similar to PHB2, cardiolipin belongs to the group of IMM mitophagy receptors. Biosynthesis of cardiolipin occurs in the IMM, where it is primary located. In response to mitochondrial stress, cardiolipin migrates to the OMM setting up a signaling platform for mitophagy and apoptosis initiation. Furthermore, migration of cardiolipin on the OMM is essential for its direct binding of with LC3 and mitophagy stimulation (53). A recent study in yeast showed that both the mitogen-activated protein kinase and the protein kinase C (PKC) are involved in cardiolipin-mediated mitophagy. Interestingly, activation of PKC was sufficient to reverse mitophagy defects phenotypes in cardiolipin-depleted cells (54). Taken together, detail mechanistic insights relative to the activation and function of IMM mitophagy receptors will provide novel therapeutics targets in numerous mitochondrial disorders.

## The Interplay Between Mitophagy and Xenophagy

The bacterial origin of mitochondria is a result of an endosymbiotic event that happened billions of years ago. Although the evolutionary changes, mitochondria retained several vestiges of their prokaryotic ancestors. First, mitochondria are semi-autonomous organelles that could expand or shrink their population through fission/fusion events independently of cell division (75). Mitochondria contain their own circular genome that displays evident bacterial characteristics such as decreased methylation events, lack of histones, polycistronic, and intron-less genetic loci (76). Furthermore, mitochondrial inner membrane is composed of cardiolipin, a specific phospholipid that exist uniquely in prokaryotic membranes (77). In addition, mitochondrial protein translation begins with *N*-formylmethionine, which is a derivative of methionine and a common feature of bacterial and organellar protein synthesis (78). Given the ancestral similarities between mitochondria and bacteria, it is worthwhile to investigate the common molecular mechanisms that regulate mitophagy and microbe selective autophagy, known as xenophagy.

The function of innate immunity is driven by the recognition of endogenous and exogenous signals by innate immune system receptors, such as toll-like receptors (TLRs), formyl peptide receptors, nucleotide oligomerization domain-like receptors (NLRs), retinoic acid-inducible gene 1 (RIG-1)-like receptors (RLRs), C-type lectin receptors (CLRs), and inflammasomes. Both pathogen-associated molecular pattern (PAMP) molecules, which are microbial derived stimulators (e.g., microbial nucleic acids, lipoproteins, and carbohydrates), and damage-associated molecular pattern (DAMPs) molecules, which are released by the cells of the host in response to injury or necrotic cell death (e.g., mtDNA, cardiolipin, ATP, and formyl peptides), are recognized by the immunity receptors mediating, in turn, inflammatory signaling pathways (10, 11).

**Figure 2:**
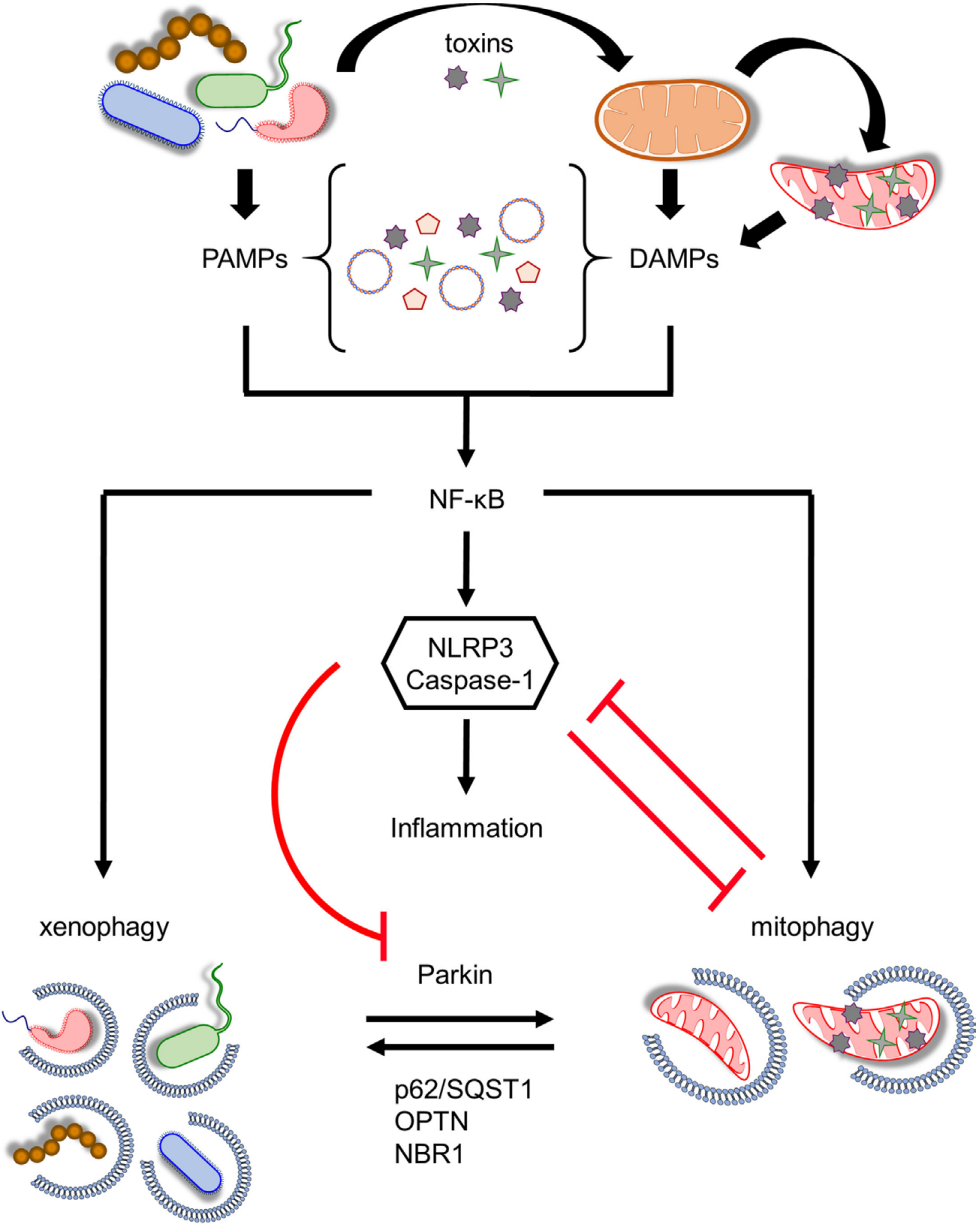
Coordination between mitophagy and xenophagy during infection. Pathogen-derived toxins and secreted proteins impair mitochondrial homeostasis leading to mitochondrial DNA and formyl peptides release and excessive generation of mitochondrial ROS. Consequently, pathogen-associated molecular patterns (PAMPs) and damage-associated molecular patterns (DAMPs) trigger nuclear factor-κB (NF-κB) transcription factor promoting immune responses through NLR family, pyrin domain-containing 3 (NLRP3) inflammasome activation and pro-inflammatory cytokines production. In parallel, NF-κB establishes a self-limiting program to prevent persistent and uncontrolled inflammation by augmenting mitophagy and pathogen removal *via* xenophagy. NLRP3 stimulation amplifies mitochondrial defects by inhibiting mitophagy through the direct caspase-1-mediated proteolytic cleavage of Parkin. Mitochondrial and bacterial autophagic processes share several common regulatory factors, including Parkin, p62/SQST1, optineurin (OPTN), and NBR1 among others, highlighting their tight communication. This intricate interplay between energy metabolism and innate immune responses upholds cellular and tissue homeostasis and survival during pathogen invasion.

Similar to mitophagy induction, PAMPs promote the recruitment of autophagic machinery through a series of ubiquitination events and the stimulation of several receptor and adaptor molecules in response to pathogen invasion. Following *Mycobacterium tuberculosis* infection in macrophages, bacterial DNA is recognized by cGMP-AMP synthase/stimulator of IFN genes mediating type 1 interferon generation and xenophagy initiation (51). Galectin-8 is a cytosolic PAMPs receptor that binds *Salmonella typhimurium* and prevents its proliferation. Interestingly, galectin-8 recruits NDP52 adaptor protein in a ubiquitin-dependent manner and promotes xenophagy (79). Moreover, several autophagy adaptor proteins, including p62, OPTN, and NBR1 with a well-established role in mitophagy, bind ubiquitinated bacteria and mediate autophagosome formation (Figure 2) (50, 80, 81). Thus, ubiquitination events have an important role in the recognition and the elimination of pathogenic microorganisms bearing a strong resemblance to mitophagy (82). Strikingly, the E3-ubiquitin ligase Parkin has been shown to mediate xenophagy (83). *M. tuberculosis* ubiqutination is abolished in murine and human Parkin-depleted macrophages resulting in defective bacterial elimination (83). Furthermore, Parkin-deficient nematodes, flies, and mice are more vulnerable to multiple pathogenic bacteria, such as *M. tuberculosis, Mycobacterium marinum, S. typhimurium, Listeria monocytogenes*, and *Pseudomonas aeruginosa* (83, 84). Congruently, several polymorphisms in the *PARK2* genetic locus are correlated with enhanced susceptibility to *Mycobacterium leprae* and *S. typhimurium* in humans, highlighting the evolutionary conserved function of Parkin in innate immunity (85–87).

The question then arises: How does bacterial infection stimulate the E3 ligase activity of Parkin? During mitochondrial removal, PINK1 is stabilized on the OMM mediating Parkin translocation and activation (74). Recently, PINK1 has emerged as a critical regulator of innate immunity, as it has been shown that loss of PINK1 enhances inflammation by attenuating the levels of pro- and anti-inflammatory cytokines leading subsequently to cell death (88). Moreover, PINK1-depleted nematodes are sensitive to *P. aeruginosa* infection (84). However, the role of PINK1 in xenophagy needs further to be elucidated. An alternative candidate of Parkin activation could be the serine/threonine kinase TBK1. Interestingly, TBK1 has an essential role in mitophagy regulation, as it has been found to phosphorylate several autophagy adaptor proteins including p62, OPTN, and NDP52, enhancing cargo recognition and autophagosomal engulfment (48, 50–52). Notably, TBK1 is required for efficient identification and removal of *M. tuberculosis* and *S. typhimurium* in mammals (50, 51). Hence, TBK1 kinase could mirror PINK1 activity during infection; however, further investigation of the functional association between TBK1 and Parkin is needed.

## Mitochondria: A Signaling Hub of Innate Immune System Stimulation

Considering the structural similarities between mitochondria and bacteria, an intricate question follows: Could mitochondria be misrecognized by the innate immunity as “invaders” promoting lethal inflammatory responses? Severe physical injury and/or trauma could lead to tissue disruption and cellular damage mediating the release of mitochondrial DAMPs molecules, such as formyl peptides and mtDNA, into the host bloodstream (89). Then, the immune system is alarmed resulting in the development of systemic inflammatory response syndrome (SIRS), which is characterized by fever, increased heart rate, low blood pressure, shortness of breath, multiple organ failure, and increased lethality rates. Although, the characteristic features of SIRS resemble sepsis, an inflammatory response to severe infection, pathogenic microorganisms are need not to be present. Thus, mitochondria lie in the heart of innate immunity initiating uncontrolled immune response upon noxious stimuli.

Several studies have shown the immunogenic capabilities of defective mitochondria (90). Impaired mitochondrial metabolism results in increased mitochondrial ROS (mtROS) levels and defective ion homeostasis. Compelling evidence has been accumulated suggesting that the cytoplasmic levels of mtDNA and mtROS signaling are critical factors in innate immunity *via* inflammasome activation (16–18). The NLR family, pyrin domain-containing 3 (NLRP3) inflammasome is one of the well-studied inflammasomes protecting the cell against pathogens invasion (13). However, dysregulation of NLRP3 activity leads to chronic inflammation and the development of several pathologies, such as neurodegeneration, metabolic disorders, and sepsis (91).

Infections impair mitochondrial homeostasis mediating mtDNA release, excessive mtROS production, and subsequently inflammasome stimulation (Figure 2). NLRP3 is triggered in response to mitochondrial damage promoting caspase-1 activation. Consequently, caspase-1 generates mature interleukin (IL)-1β and IL-18 promoting inflammatory cell death (14, 92). To this direction, there is evidence suggesting that LC3B-, ATG5-, ATG16L1-, and Beclin1-deficient macrophages display accrual of defective mitochondria, increased cytosolic levels of mtDNA, and mtROS in response to noxious stimuli, resulting in NLRP3 activation and IL-1β secretion (15, 17, 18). In addition to mtDNA and mtROS, NLRP3 is also activated by cardiolipin. Mitochondrial membrane depolarization triggers the translocation of cardiolipin from the IMM to OMM promoting its direct association with NLRP3 (93). NLRP3–cardiolipin interaction is pivotal for inflammasome stimulation indicating that mitochondria act as a central signaling platform for innate immune responses. Experimental evidence highlights the existence of a positive feedback loop between inflammasome and mitochondria, since caspase-1 amplifies mitochondrial dysfunction by impairing mitochondrial membrane potential, increasing mitochondrial membrane permeabilization, and promoting mitochondrial network fragmentation to enhance inflammatory responses (19). Notably, mitophagy is also inhibited upon inflammasome stimulation, since it is reported that Parkin is cleaved by caspase-1 preventing the degradation of damaged organelles (19, 94). Concurrently, accumulation of defective mitochondria results in enhanced mtROS production and hyperstimulation of NLRP3 (Figure 2).

Nuclear factor-κB (NF-κB) is the master coordinator of inflammatory signaling acting downstream of immune receptors (95, 96). In addition to the production of multiple inflammatory chemokines and cytokines, NF-κB also regulates inflammasome activation (97). The TLR9 innate immune receptor recognizes mtDNA, which is released from necrotic cells, resulting in NF-κB nuclearization and the induction of several pro-inflammatory cytokines, such as tumor necrosis factor α (TNFα) and IL-6 (98). A recent study uncovered a self-regulatory and anti-inflammatory pathway, whereby NF-κB restricts NLRP3 function through p62-dependent mitophagy (20). NF-κB enhances the expression of p62 adaptor molecule mediating the removal of damaged mitochondria. Moreover, p62-, Parkin-, and ATG7-depleted macrophages display pronounced NLRP3 activity, since they accumulate defective organelles releasing inflammasome-activating signals in response to harmful stimuli (20). Therefore, NF-κB establishes a self-limiting program to inhibit unresolved inflammation, whereby mitophagy has a central role preventing tissue damage through the maintenance of mitochondrial metabolism (Figure 2).

**Figure 3:**
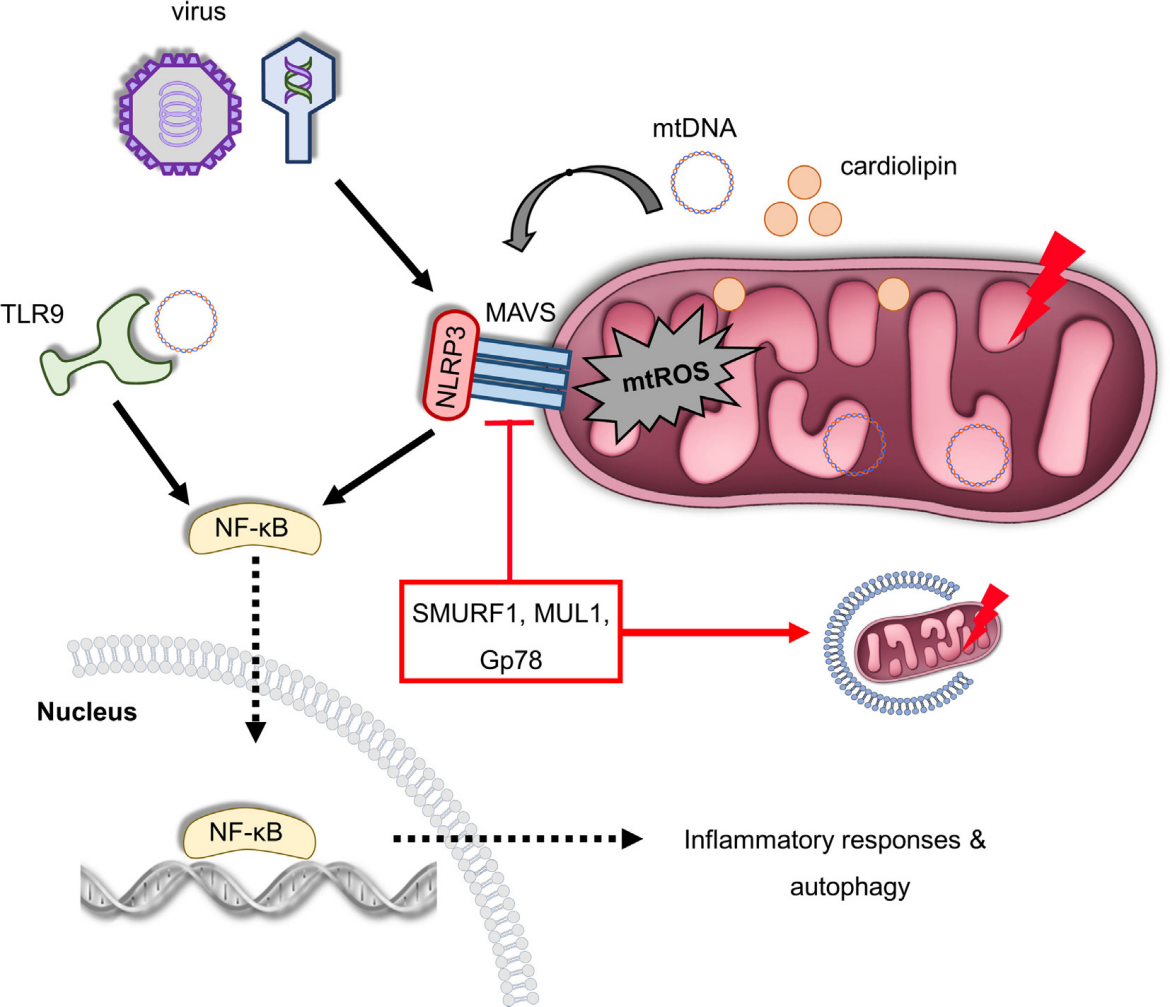
Mitochondria act as a signaling platform of innate immunity. Viral infection triggers mitochondrial antiviral signaling protein (MAVS) oligomerization in OMM, which promotes the expression of several inflammatory and immune genes *via* nuclear factor-κB (NF-κB) activation. Furthermore, MAVS can be activated by excessive mitochondrial ROS (mtROS) production recruiting NLRP3 on the OMM in response to infection and mitochondrial damage. In turn, NLRP3 assembly and activity is enhanced due to elevated reactive oxygen species levels. In addition, depolarized mitochondria release cardiolipin and mitochondrial DNA (mtDNA) in the cytosol-stimulating NLRP3, which generates pro-inflammatory cytokines and mediates inflammation. mtDNA can be also sensed by toll-like receptor (TLR) 9 immune receptors promoting activation of both NLRP3 and NF-κB. The immunosuppressive role of the ubiquitin E3 ligases SUMRF1, MUL1, and Gp78 is characterized both by the negative regulation of MAVS signaling and mitophagy stimulation. Thus, multiple innate immune signaling pathways, such as MAVS, NLRP3 inflammasome, and TLR9, depend on mitochondrial function to regulate immune responses.

Mitochondrial antiviral signaling protein (MAVS) is an RLR immune receptor that is localized on the OMM (99). Elevated mtROS levels trigger oligomerization of MAVS, which subsequently activate NF-κB to regulate host defense and inflammation (100). Interestingly, MAVS recruits NLRP3 on the OMM during viral infection (Figure 3). Thereby, inflammasome assembly and activity is enhanced due to the close proximity of NLRP3 with the sites of mtROS generation (101, 102). MAVS signaling is negatively regulated by ubiquitination events mediated by the ubiquitin E3 ligases SMURF1, Gp78, and Mul1 (103–105). Notably, SMURF1, Gp78, and MUL1 are also involved in the regulation of mitochondrial removal indicating the immunosuppressive role of mitophagy in response to noxious stimuli (Figure 3) (106–108). Indeed, a very recent study revealed that anti-inflammatory cytokine IL-10 promotes mitophagy to restrain inflammasome activity and the uncontrolled inflammatory responses upon lipopolysaccharide (LPS) treatment (109).

Altogether, these results demonstrate the pivotal role of mitochondria in the innate immune signaling pathways and underline mitophagy as a key regulatory mechanism limiting excessive inflammation and preserving tissue homeostasis. Although the delineation of mitophagy–innate immunity interplay represents a milestone in the field of immunometabolism, several mechanistic questions still remain elusive, including how mitophagy and inflammasome activity are coordinated in response to infection, physical injury, and/or trauma and which already known mitophagy factors are involved during mitochondrial removal in immune cells.

## Mitophagy and Macrophage Homeostasis

**Figure 4:**
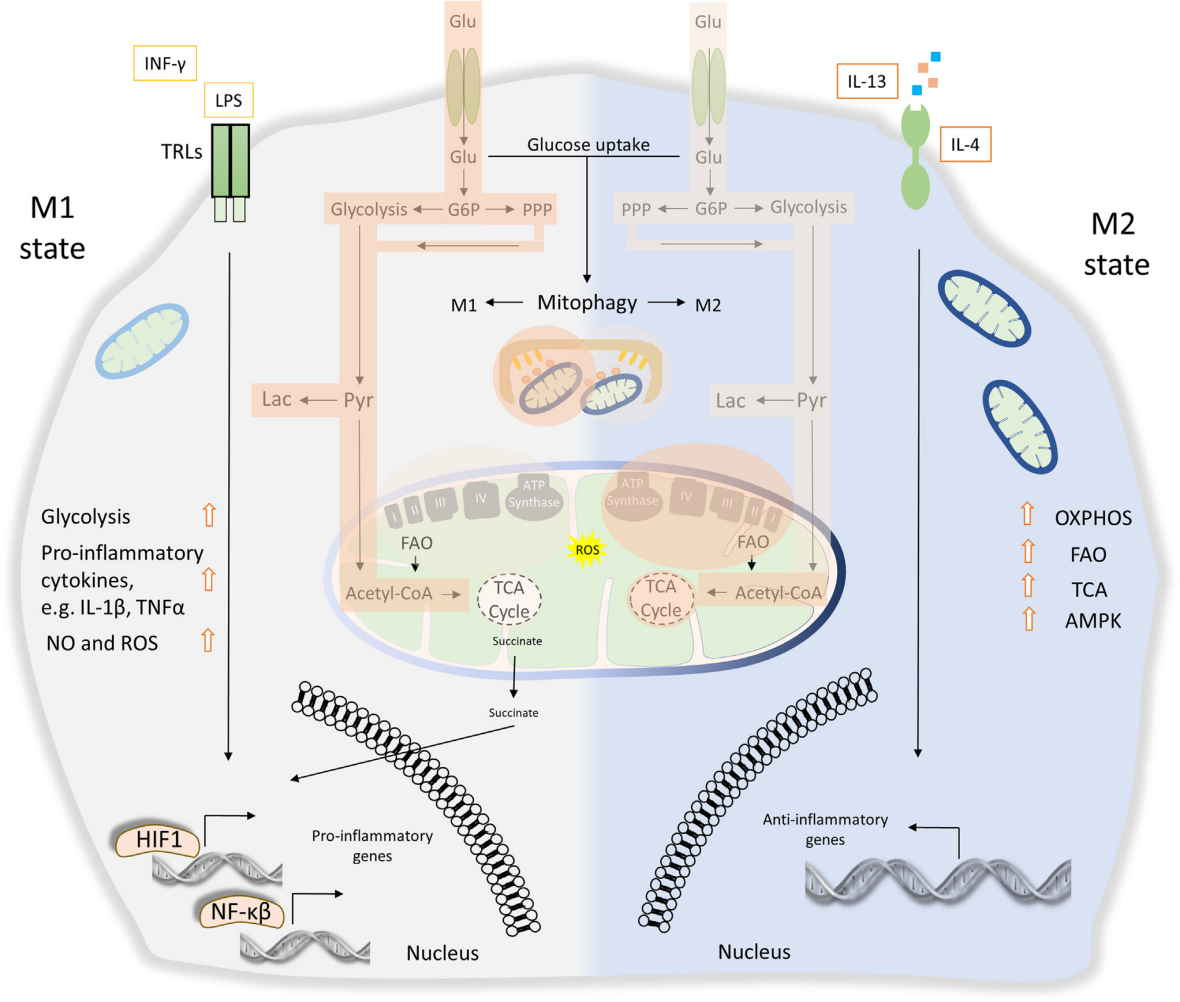
The role of energy homeostasis and mitophagy in metabolic signature of M1 and M2 polarization. M1 macrophages are characterized by secretion of pro-inflammatory cytokines, such as interleukin (IL)-1β and tumor necrosis factor α (TNFα). Thus, M1 macrophages display high tumoricidal and microbicidal properties. M1 macrophages rely heavily on glycolysis accompanied with increased glucose uptake and pentose phosphate pathway (PPP). Metabolic byproducts, such as reactive oxygen species (ROS) and nitric oxide (NO), are abundantly produced. During M1 macrophage polarization, succinate is released to the cytosol and promotes stabilization of hypoxia-inducible factor 1 (HIF1), which in turn drives the transcription of several genes involved in glycolysis. Translocation of both HIF1 and nuclear factor-κB (NF-κB) to the nucleus mediates the expression of numerous pro-inflammatory genes. On the other hand, anti-inflammatory function of M2 macrophages relies more on mitochondrial oxidative phosphorylation (OXPHOS), tricarboxylic cycle (TCA) fluxes, and fatty acid oxidation (FAO). AMP-activated protein kinase (AMPK) is activated and triggers FAO, which fuels OXPHOS. Mitophagy act as a key factor of M1/M2 differentiation. During M1 polarization, mitochondria clearance through mitophagy stimulation favors metabolic reprogramming to glycolysis. Activity of glycolysis, OXPHOS, TCA, and FAO is labeled with bright orange frames (less active state) and dark orange frames (more active state).

Macrophages are indispensable phagocytic cells coordinating both pro-inflammatory and anti-inflammatory responses as wells as tissue homeostasis and repair during infection (Figure 4) (110). Halted macrophages can be stimulated and adapted to the host and pathogen nature while their activity undergoes dynamic changes (111). Following pathogen invasion and recognition through the innate immune receptors, such as TLRs and CLRs, immune cells produce inflammatory cytokines (112–114). Particularly, secretion of interferon γ (IFNγ) by helper T cells 1, triggers macrophages pro-inflammatory polarization, known as classically activated or M1 macrophages (115, 116). Similar to IFNγ production, LPS signaling also stimulates M1 macrophages through TLRs (117). The tumoricidal and microbicidal properties of M1 macrophages are highlighted by their ability to produce and release several pro-inflammatory cytokines, such as IL-1β and TNFα, and cellular byproducts including ROS and nitric oxide (NO) (118–120). On the contrary, polarization of macrophages toward an anti-inflammatory phenotype is known as alternatively activated or M2 macrophages. In response to specific stimulus, a large spectrum of mediators has been reported to activate M2 macrophages (121). Stimulation of M2 macrophages requires in part, secretion of IL-4 and IL-13 cytokines through Th2 cells (122, 123). Particularly, IL-4- and IL-13-induced M2 macrophages are important for wound healing, while IL-10-induced M2 macrophages regulating host immunity and tissue homeostasis (124). In addition, the secretion of immune complexes together with agonist of TLRs triggers M2 macrophages exerting an immunoresponsive function (125). Non-activated macrophages could be differentiated to M2 phenotype by the transforming growth factor-β activity (124, 126). Given the distinct modes of action, further classification of M2 macrophages has been proposed (57, 115, 127).

Apparently, numerous pathogen-derived molecules and biochemical signals orchestrate macrophage activation from pro-inflammatory M1 toward to anti-inflammatory M2 phenotype, including their intermediate responses (110). As a consequence of these extreme heterogeneities, M1 and M2 macrophages undergo broad transcriptional and metabolic alternations, beyond their energy demands (8). Metabolic signature of both M1 and M2 macrophages activation imposes a tight coordination between glycolysis, pentose phosphate pathway (PPP), fatty acid oxidation (FAO), mitochondrial oxidative phosphorylation (OXPHOS), and tricarboxylic cycle (TCA) fluxes (128). It has been shown that M1 macrophages activity is mostly affected by glycolysis and PPP while mitochondrial OXPHOS and TCA capacities are decreased (129, 130). Specifically, LPS-activated M1 macrophages promote the release of succinate dehydrogenase to the cytosol resulting in the stabilization of HIF1, which in turn regulates the expression of several pro-inflammatory genes such as IL-1β (Figure 4). Absence of HIF1 or inhibition of glycosis in bone marrow-derived macrophages (BMDMs) failed to induce LPS-mediated IL-1β expression (131). Metabolic rewiring from OXPHOS toward glycolysis followed by IL-1β induction requires the stimulation of pyruvate kinase M2 in LPS-activated M1 macrophages (132). On the other hand, M2 macrophages activity relies more on FAO, mitochondrial OXPHOS, and TCA, but less in glycolysis and PPP fluxes (133). Particularly, the stimulation of M2 macrophage requires AMP-activated protein kinase activation as well as induction of FAO to fuel OXPHOS (Figure 4) (134). Notably, byproducts of mitochondrial metabolism, such as mtROS, have also been involved in innate immune responses and macrophages activity. Production of mtROS has been shown to mediate inflammatory cytokine secretion (135). Accordingly, several studies suggest that augmented mtROS levels are required for the bactericidal activity of macrophages (119, 120).

Only recently, the metabolic signature underpinning macrophage activation has been associated with mitochondrial clearance through mitophagy. A latter report suggests that NIX-mediated mitophagy regulates metabolic shift during macrophage differentiation (136). Mitophagy is triggered during M1 macrophage polarization in response to LPS/IFNγ treatment favoring metabolic rewiring to glycolysis. Interestingly, NIX-depleted M1 macrophages present decreased levels of glycolytic enzymes and pro-inflammatory cytokines, indicating metabolic defects during their differentiation process (136). IFN-stimulated gene 15 (ISG15) has also been shown to regulate both cellular metabolism and mitophagy of BMDMs in response to vaccinia virus infection (137). In addition to impaired mitochondrial function, ISG15-deficient macrophages present reduced Parkin protein levels and inhibition of mitophagy upon IFNγ stimulation. Moreover, loss of ISG15 leads to defective macrophages polarization and subsequently to enhanced virus susceptibility (137).

As reported, IL-10-depleted murine BMDMs favors glucose uptake and glycolysis while inhibits OXPHOS in response to LPS treatment (109). IL-10-deficient macrophages display accumulation of damaged mitochondria due to inhibition of mitophagy. Interestingly, IL-10 regulates mitochondrial homeostasis through the inhibition of mTOR signaling (109). A recent study in mouse macrophages has also demonstrated that high glucose supplementation results in mitophagy defects and promotes M1 macrophages activation (138). Therefore, mitophagy regulation is indispensable for the proper determination of M1/M2 macrophage phenotypes (Figure 4). Recently, the essential role of fine-tuned mitochondrial metabolism and mitophagy was underlined in a mouse model of sepsis (139). Bone marrow-derived mesenchymal stem cells (BMSCs) promote survival and performance of various organs during septic shock. It is shown that the beneficial effects of BMSCs were mediated by mitophagy induction in cocultured BMDMs resulting in decreased mtROS levels and inflammasome restriction during cecal ligation and puncture-induced sepsis (139). Although the beneficial effect of mitophagy induction during pronounced inflammatory conditions, mitophagy hyperstimulation could also be detrimental for cellular physiology. Runaway mitophagy mediates mitochondrial content elimination conferring resistance to apoptosis in alveolar macrophages and subsequently leads to the development and progression of idiopathic pulmonary fibrosis (IPF) (140).

Taken together, mitochondrial homeostasis and mitophagy are crucial for the determination of macrophages functional behavior. However, the mechanistic details that orchestrate macrophage intracellular metabolism remain still elusive. A better understanding of the interconnection between mitophagy and macrophages fate and function in response to injury and/or infection could lead to unpreceded understanding of several immune disorders.

## Mitochondrial Function and Mitophagy in Septic Shock

Infection triggers the activation of immune system promoting the production and release of several cytokines and chemokines into the host circulation. In turn, inflammatory responses are initiated mediating the signal throughout the body of the organism to confer protection against pathogens. However, persistent systemic inflammatory conditions, such as sepsis, impairs cellular metabolism leading to generalized shock, compromised function of multiple organs and eventually to death. Sepsis or septicemia is a life-threatening condition and a leading cause of morbidity and mortality worldwide (141–143). The pronounced mitochondrial defects, which are described in septic conditions, and the significant role of mitochondria in innate immune signaling indicate their involvement in the development and progression of sepsis (144).

Peripheral mononuclear blood cells, isolated form septic patients, present hyperactivated mitogen-activated protein (MAP) kinase kinase 3 (MKK3) (145). Notably, MKK3 stimulates p38 MAP kinase signaling to promote septic shock (146, 147). MKK3-depleted macrophages display improved energy metabolism, which is characterized by reduced mtROS production, larger and elongated mitochondria, elevated membrane potential and ATP generation during LPS challenge (148, 149). These results suggest that MKK3 alters mitochondrial function to further enhance inflammatory responses. Indeed, MKK3 depletion restricts NF-κB nuclearization and inflammasome stimulation conferring resistance to septic injury (147, 148). Recently, MKK3 has revealed as an essential factor of mitochondrial homeostasis, since MKK3 deficiency influences the modulation of several proteins, including sirtuin 1, PINK1, and Parkin among others, to promote both the induction of mitophagy and mitochondrial biogenesis (145).

Further supporting the immunosuppressive role of mitophagy in sepsis, a recent study showed that senstrin 2 (SESN2) restrains NLRP3 activity by promoting the elimination of damaged mitochondria in macrophages (150). Interestingly, SESN2 mediates the association between p62 and the ubiquitin chains on the OMM, thereby, promoting the perinuclear localization of dysfunctional organelles. In turn, SESN2 initiates autophagosomal formation and mitochondrial turnover by increasing the levels of the autophagy initiator protein ULK1 (150). It has been reported that NO prevents NLRP3 activation and protects against LPS-induced septic shock (151). Notably, NO, generated by nitric oxide synthase 2, upregulates SESN2 protein levels contributing to inflammasome suppression during LPS-induced sepsis (150). In addition, basal levels of NO could also promote mitochondrial translocation of Parkin mediating PINK1-independent mitophagy (152). On the other hand, under nitrosative stress conditions, the excessive NO generation induces *S*-nitrosylation of PINK1 inhibiting its kinase activity and preventing Parkin mitochondrial recruitment (153). Thus, further investigation is needed to delineate the role of NO activity in the regulation of mitophagy and energy metabolism in immune cells.

## Concluding Remarks

Mitophagy holds an essential role in the regulation of inflammatory responses. Several molecular mechanisms are coordinated to mediate mitophagy, preserving cellular and organismal survival in response to intracellular and environmental stimuli (68). Mitophagy deregulation leads to impaired mitochondrial metabolism and eventually to systemic unresolved inflammation and tissue collapse. While basal changes in mitochondrial number and function induce mitophagy, under severe mitochondrial damage excessive mitophagy leads to programmed cell death (154). Therefore, mitophagy impacts organismal health and disease in a context- and dose-dependent fashion (66, 140, 155, 156). To this direction, shortage of mitochondrial population due to induction or persistent mitophagy has also been reported (140, 157, 158). It is becoming evidence that mitophagy defects as well as excessive mitophagy events represent common features of several pathologies. In particular, runaway mitophagy lowers mitochondrial population in alveolar macrophages conferring resistance to apoptosis, which in turn, leads to IPF progression (140). As noted, growth of tumor cells upon hypoxia requires stimulation of glycolysis and lactate production accompanied by increased mitophagy (159, 160). Within this scope, mitochondrial localization of valosin-containing protein drives hyperactivation of mitophagy and leads to neurodegeneration in Huntington’s disease (161). Overall, the degree to which mitophagy contributes to these pathologies has not been elucidated yet. Moreover, it remains to be clarified whether pharmacological stimulation or inhibition of mitophagy represents a potent therapeutic strategy for several pathologies including immune disorders, cancer, and neurodegeneration among others. Although great progress has been already made in the field of mitophagy-inducing drugs, better understanding of the molecular mechanisms would ensure the identification of novel targets for maximum therapeutic efficiency.

Several synthetic and/or natural compounds have been shown to induce mitophagy maintaining cellular and organismal homeostasis (162). p62/SQST1-mediated mitophagy inducer (PMI) is a chemical compound that promotes mitochondrial removal through nuclear factor E2-related factor 2 (Nrf2) stimulation (163). Nrf2 is the master regulator of cellular homeostasis orchestrating the gene expression of multiple cytoprotective proteins, including antioxidant, anti-inflammatory, and detoxification enzymes among others, to enhance survival and viability during stress (164). Moreover, Nrf2 activity is pivotal for proper mitochondrial function and metabolism, since it regulates the expression levels of several mitochondrial related genes (165). PMI prevents the proteasomal degradation of Nrf2 by disrupting its association with Kelch-like ECH-associated protein 1 (163). In turn, Nrf2 is stabilized and enhances p62/SQST1 expression promoting PINK1/Parkin-independent mitophagy upon PMI supplementation (163). Notably, deregulation of Nrf2 function results in the development of autoimmune diseases and increased susceptibility to pathogens, since Nrf2 is implicated in several innate immune responses (166–169). Therefore, PMI administration could be beneficial against the aforementioned pathological conditions.

Natural occurring compounds able to induce mitochondrial turnover have attracted much attention in recent years. As such, spermidine and urolithin A have been shown to induce mitochondrial elimination promoting longevity and stress resistance in many organisms, including mice, flies, and nematodes (170–172). In addition to mitophagy, both spermidine and urolithin A present anti-inflammatory properties modulating mitochondrial metabolism and subsequently NF-κB activity (173–178).

The balanced interplay between the inflammatory and the immunosuppressive signaling pathways highlights that sustaining mitochondrial network integrity and energy metabolism safeguard tissue and organismal homeostasis in response to constant exposure to immunogenic signals. Hence, examination of already FDA-approved drugs and several pharmacological screenings are taking place to characterize novel molecules that can be used to enhance immune system homeostasis through the regulation of mitophagy. Although, experimental evidence underlines the cytoprotective effects of mitophagy modulators on animal disease models, the therapeutic potential and the levels of cytotoxicity on humans remain to be determined. Thus, interventional clinical studies need to be organized to monitor and validate the therapeutic capacity of mitophagy-inducing agents against immune system diseases.

## Author Contributions

IG and KP wrote the manuscript. NT organized and edited the manuscript.

## Conflict of Interest Statement

The authors declare that the research was conducted in the absence of any commercial or financial relationships that could be construed as a potential conflict of interest.
